# Comparison of Evaluations for Heart Transplant Before Durable Left Ventricular Assist Device and Subsequent Receipt of Transplant at Transplant vs Nontransplant Centers

**DOI:** 10.1001/jamanetworkopen.2022.40646

**Published:** 2022-11-07

**Authors:** Thomas M. Cascino, Jeffrey S. McCullough, Xiaoting Wu, Michael J. Pienta, James W. Stewart, Robert B. Hawkins, Alexander A. Brescia, Ashraf Abou el ala, Min Zhang, Pierre-Emmanuel Noly, Jonathan W. Haft, Jennifer A. Cowger, Monica Colvin, Keith D. Aaronson, Francis D. Pagani, Donald S. Likosky

**Affiliations:** 1 Division of Cardiovascular Medicine, University of Michigan Medical School, Ann Arbor; 2 Department of Cardiac Surgery, University of Michigan Medical School, Ann Arbor; 3 Department of Biostatistics, School of Public Health, University of Michigan, Ann Arbor; 4 Department of Health Management and Policy, School of Public Health, University of Michigan, Ann Arbor; 5 Division of Cardiovascular Medicine, Henry Ford Hospital, Detroit, Michigan

## Abstract

**Importance:**

In 2020, the Centers for Medicare & Medicaid Services revised its national coverage determination, removing the requirement to obtain review from a Medicare-approved heart transplant center to implant a durable left ventricular assist device (LVAD) for bridge-to-transplant (BTT) intent at an LVAD-only center. The association between center-level transplant availability and access to heart transplant, the gold-standard therapy for advanced heart failure (HF), is unknown.

**Objective:**

To investigate the association of center transplant availability with LVAD implant strategies and subsequent heart transplant following LVAD implant before the Centers for Medicare & Medicaid Services policy change.

**Design, Setting, and Participants:**

A retrospective cohort study of the Society of Thoracic Surgeons Intermacs multicenter US registry database was conducted from April 1, 2012, to June 30, 2020. The population included patients with HF receiving a primary durable LVAD.

**Exposures:**

LVAD center transplant availability (LVAD/transplant vs LVAD only).

**Main Outcomes and Measures:**

The primary outcomes were implant strategy as BTT and subsequent transplant by 2 years. Covariates that might affect listing strategy and outcomes were included (eg, patient demographic characteristics, comorbidities) in multivariable models. Parameters for BTT listing were estimated using logistic regression with center-level random effects and for receipt of a transplant using a Cox proportional hazards regression model with death as a competing event.

**Results:**

The sample included 22 221 LVAD recipients with a median age of 59.0 (IQR, 50.0-67.0) years, of whom 17 420 (78.4%) were male and 3156 (14.2%) received implants at LVAD-only centers. Receiving an LVAD at an LVAD/transplant center was associated with a 79% increased adjusted odds of BTT LVAD designation (odds ratio, 1.79; 95% CI, 1.35-2.38; *P* < .001). The 2-year transplant rate following LVAD implant was 25.6% at LVAD/transplant centers and 11.9% at LVAD-only centers. There was an associated 33% increased rate of transplant at LVAD/transplant centers compared with LVAD-only centers (adjusted hazard ratio, 1.33; 95% CI, 1.17-1.51) with a similar hazard for death at 2 years (adjusted hazard ratio, 0.99; 95% CI, 0.90-1.08).

**Conclusions and Relevance:**

Receiving an LVAD at an LVAD-transplant center was associated with increased odds of BTT intent at implant and subsequent transplant receipt for patients at 2 years. The findings of this study suggest that Centers for Medicare & Medicaid Services policy change may have the unintended consequence of further increasing inequities in access to transplant among patients at LVAD-only centers.

## Key Points

**Question:**

Is the presence of a heart transplant program associated with differential evaluation for transplant or transplant among patients who receive a left ventricular assist device (LVAD)?

**Findings:**

In this cohort study of 22 221 LVAD recipients from the Society of Thoracic Surgeons Intermacs database, patients receiving durable LVAD at centers that also performed heart transplants were significantly more likely to receive an LVAD as a bridge to transplant. In addition, patients treated at a combined LVAD/transplant center were more likely to receive a heart transplant in the subsequent 2 years.

**Meaning:**

The findings of this study suggest that the increased use of LVAD at centers that do not perform transplants has the potential to contribute to inequities in access to heart transplant, the gold-standard therapy for advanced heart failure.

## Teaser

This cohort study examines access to heart transplant in patients at centers that implant left ventricular assist devices but do not perform transplants vs those at centers that provide both left ventricular assist devices and transplant.

## Introduction

Heart failure (HF) refractory to medical therapy affects approximately 300 000 people in the US and is associated with up to a 75% 1-year mortality.^1,2,3,4,5,6^ Advanced therapies, including heart transplant and durable left ventricular assist device (LVAD) implant, are life saving for patients with advanced HF, with 1-year survival for both LVAD and transplant greater than 85%.^7,8^ In December 2020, the Centers for Medicare & Medicaid Services (CMS) implemented a national coverage decision that included relaxed restrictions at LVAD-only implanting centers.^9^ These changes included removing (1) the therapeutic intent, (2) the requirement that the patient be active on the waiting list maintained by the Organ Procurement and Transplantation Network when receiving a durable LVAD as a bridge to transplant (BTT), and (3) the requirement that the implanting site, if different than the Medicare-approved transplant center, must receive written permission from the Medicare-approved transplant center before implantation of a BTT LVAD.^9^

Although there is enthusiasm for the change, given the potential to make durable LVADs more accessible, the association between the coverage change and access to heart transplant, the gold-standard therapy for advanced HF,^10^ is uncertain. On the one hand, it is plausible that removing the requirement for determination of transplant ineligibility by a Medicare-approved heart transplant center may facilitate access to durable LVAD therapy for appropriate patients while transplant-eligible patients will continue to be referred to transplant centers for consideration for transplant. On the other hand, by removing the coevaluation between Medicare-approved transplant programs and nontransplant programs, there is a potential unintended consequence of transplant-eligible patients not receiving a transplant.

This large-scale observational cohort study evaluated the association of transplant availability at a center before the CMS policy change with its national coverage decision. Specifically, this study examined the association between LVAD center transplant availability and therapeutic intent and subsequent heart transplant following durable LVAD implant. It was hypothesized that there would be a greater likelihood of durable LVAD implant as BTT and receipt of a heart transplant after durable LVAD implant at Medicare-approved transplant centers compared with non–Medicare-approved transplant centers (LVAD-only centers). This study’s findings may provide insight into the potential association between the CMS policy change and equitable access to transplant for patients receiving care at nontransplant centers.

## Methods

### Data Sources and Sample

The Society of Thoracic Surgeons (STS) Intermacs database was the primary data source (additional details in eMethods 1 in Supplement 1). Center-level transplant status by year was determined by publicly available data from the Organ Procurement and Transplantation Network.^11^ Hospital characteristics were abstracted from the American Hospital Association Annual Survey from 2012.^12^ Transplant volume and center characteristics were linked to patient-level STS Intermacs data by the data coordinating center at The University of Alabama at Birmingham. The deidentified analytical data set was created through the STS Participant User File program. This study was approved as not regulated owing to the use of deidentified data by the University of Michigan Institutional Review Board and followed the Strengthening the Reporting of Observational Studies in Epidemiology (STROBE) reporting guideline.

The study population included all adults undergoing de novo primary durable continuous-flow LVAD implantation between April 1, 2012, and June 30, 2020. The start date was based on the availability of patient-level information in STS Intermacs, version 4.0. Patients were excluded if undergoing LVAD exchange, had a concurrent right VAD, or were missing data on either age or implantation center.

### Outcomes

The prespecified primary outcomes were use of BTT therapeutic intention to treat and receipt of heart transplant within 2 years following LVAD implant. The initial therapeutic intention-to-treat strategy was categorized as BTT, bridge to candidacy for transplant, and destination therapy (full details described in eMethods 2 in Supplement 1).^13,14^

### Statistical Analysis

This study evaluated the association between Medicare-approved transplant center status for an LVAD implant center for both initial BTT strategy and receipt of transplant at 2 years. The primary independent variable of interest was center Medicare-approved heart transplant status (LVAD/transplant or LVAD only), determined by whether a center performed any heart transplants during the year in which an LVAD was placed. As such, centers that started or stopped performing transplants would have patients in both groupings.

Continuous variables are displayed as median (IQR). Univariable comparisons were performed using the Fisher exact test for categorical variables, Wilcoxon rank sum test for continuous variables or zero-inflated Poisson regression. Trends in the number of LVAD/transplant and LVAD-only centers and LVADs implanted by each center type were determined by linear regression.

Random-effects logistic regression was used to measure associations between center status and BTT strategy intent at implantation accounting for unobserved center-level effects. Results are presented as odds ratios and estimated marginal effects (ie, the percentage of estimated BTT use by center type). Candidate variables that could be associated with both the eligibility for and receipt of transplant (patient demographic characteristics, clinical characteristics [eg, comorbidities], and hospital data [eg, bed size]) were selected by HF experts for model inclusion (eTable 1 in Supplement 1). Race was reported by sites and categorized as American Indian or Alaska Native, Asian, African American or Black, Hawaiian or other Pacific Islander, White, Unknown/Undisclosed, or Other/none of the above. Due to low numbers in groups, race was categorized as White, Black, or other in this analysis. The STS method for imputation was used for missing variables with categorical variables assumed to have the lowest risk category and continuous variables imputed to the mean.^15^ Variables with less than 5% missingness were candidate variables, with the exception of right atrial pressure, which had 32.7% missingness.^15^ Backward selection using a *P* value cutoff threshold of <.001 was used to select variables in the final multivariable model. The model selection process excluded the variable of interest—center status—to enable inference about the association of center status after model selection,^16^ with a priori inclusion in the final model. A sensitivity analysis was performed to determine whether transplant center volume was associated with listing strategies with centers categorized into volume categories based on the number of transplants performed in the year of LVAD implant (0 transplants, 1-9 transplants, 10-19 transplants, 20-29 transplants, 30-39 transplants, 40-49 transplants, 50-59 transplants, and >60 transplants).

Time-to-event analyses accounting for competing events were used to assess the association between center status and receipt of transplant within 2 years following LVAD implant. First, the cumulative incidences for transplant and death by center status were determined. Next, a multivariate cause-specific Cox proportional hazards regression model was performed with variable selection for the primary outcome of transplant in the 2 years following LVAD with death as a competing event and censoring at last follow-up. With the association of transplant center status and listing strategy known, this analysis was performed with variable selection without device strategy included. A robustness analysis was performed to examine the interaction between transplant center status and device strategy. Sensitivity analyses included (1) adjusting for device strategy (eg, BTT or destination therapy), (2) limiting the sample to recipients younger than 70 years, (3) using a *P* value cutoff of <.10 to select variables in the final multivariable model, (4) not including right atrial pressure as a candidate variable, (5) testing whether the association between center status and transplant varied by device strategy, and (6) reestimating the model using a propensity score–matching method. The propensity for receiving care at an LVAD/transplant center was estimated using variables associated with listing strategies and transplant eligibility (eg, device strategy, patient demographic characteristics, comorbidities, region, and year) (eTable 2 in Supplement 1). Patients were matched 1:1 using the greedy matching method.^17^ A 2-tailed *P* ≤ .05 was considered statistically significant.

## Results

**Figure 1.**
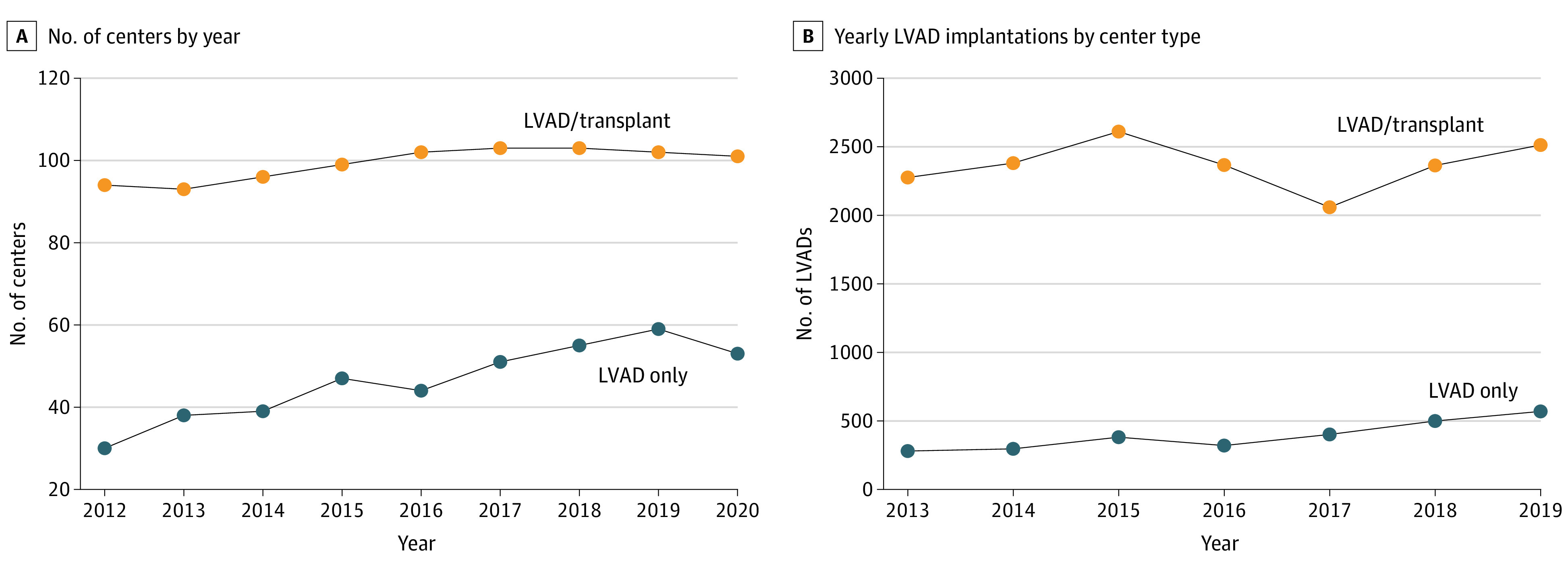
Number of Centers and Left Ventricular Assist Devices (LVADs) Performed by Center Type by Year A, The number of centers implanting LVADs increased for both LVAD-only (*P* < .001 for trend) and LVAD/transplant centers (*P* = .001 for trend) from 2012 to 2020. B, The number of LVADs implanted at LVAD-only centers increased in the study period (*P* = .003 for trend), but there was no significant change in volume (*P* = .91 for trend) at LVAD/transplant centers from 2013 to 2019, the years in which 365 days of data available.

In total, 22 221 patients with a median age of 59.0 (IQR, 50.0-67.0) years, of whom 17 420 (78.4%) were men and 4801 were women (21.6%), underwent primary durable continuous-flow LVAD implant from April 1, 2012, to June 30, 2020 (Table 1; eFigure in Supplement 1). Table 1 displays the baseline characteristics of recipients by center status, with 14.2% (n = 3156) receiving a durable LVAD at an LVAD-only center. Recipients at an LVAD-only center were older and more likely to be admitted for LVAD placement. LVAD-only centers had fewer beds, were more likely to be in cities outside of the largest 100 cities in the US, and were less likely to be affiliated with a medical school or have graduate medical education than LVAD/transplant centers (eTable 3 in Supplement 1). The number of LVAD-only centers (*P* < .001 for trend) and LVAD procedures performed (*P* = .003 for trend) at LVAD-only centers increased during the study period (Figure 1). There was a smaller increase in the number of LVAD/transplant centers (*P* = .001 for trend), without an increase in LVAD implants at such centers from 2013 to 2019 (*P* = .91 for trend).

**Table 1.** Baseline Characteristics of LVAD Recipients

Characteristic	No. (%)			*P* value^a^
	Overall (N = 22 221)	LVAD only (n = 3156)	LVAD/transplant (n = 19 065)	
Demographic characteristics				
Age, median (IQR), y	59.0 (50.0-67.0)	63.5 (54.0-71.0)	59.0 (49.0-66.0)	<.001
Sex				
Male	17 420 (78.4)	2472 (78.3)	14 948 (78.4)	.94
Female	4801 (21.6)	684 (21.7)	4117 (21.6)	
Blood type				
O	10 699 (48.1)	1474 (46.7)	9225 (48.4)	.32
A	7925 (35.7)	1166 (36.9)	6759 (35.5)	
B	2842 (12.8)	411 (13.0)	2431 (12.8)	
AB	755 (3.4)	105 (3.3)	650 (3.4)	
Race^b^				
Black	5857 (26.4)	791 (25.1)	5066 (26.6)	.10
White	14 385 (64.7)	2096 (66.4)	12 289 (64.5)	
Other	1979 (8.9)	269 (8.5)	1710 (9.0)	
Hispanic ethnicity	1492 (6.7)	188 (6.0)	1304 (6.8)	.07
BMI, median (IQR)	27.9 (24.1-32.5)	27.7 (23.9-32.5)	27.9 (24.1-32.5)	.89
Educational level				
None/unknown	5995 (27.0)	858 (27.2)	5137 (26.9)	.12
Greater than high school	8727 (39.3)	1281 (40.6)	7446 (39.1)	
High school	7499 (33.7)	1017 (32.2)	6482 (34.0)	
Marital status				
Single	4501 (20.3)	545 (17.3)	3956 (20.8)	<.001
Married	13 390 (60.3)	1937 (61.4)	11 453 (60.1)	
Divorced/separated or other	4330 (19.5)	674 (21.4)	3656 (19.2)	
Payer				
Medicare	4686 (21.1)	983 (31.1)	3703 (19.4)	<.001
Medicaid	1398 (6.3)	202 (6.4)	1196 (6.3)	
Commercial and HMO	3138 (14.1)	472 (15.0)	2666 (14.0)	
Other	12 999 (58.5)	1499 (47.5)	11 500 (60.3)	
Clinical characteristics				
Device type				
Axial	10 898 (49.0)	1832 (58.0)	9066 (47.6)	<.001
Hybrid magnetically levitated	6485 (29.2)	432 (13.7)	6053 (31.7)	
Fully magnetically levitated	4838 (21.8)	892 (28.3)	3946 (20.7)	
STS Intermacs Patient Profile				
1	3562 (16.0)	366 (11.6)	3196 (16.8)	<.001
2	7716 (34.7)	1072 (34.0)	6644 (34.8)	
3	7804 (35.1)	1249 (39.6)	6555 (34.4)	
4-7	3139 (14.1)	469 (14.9)	2670 (14.0)	
NYHA functional class				
I	22 (0.1)	2 (0.1)	20 (0.1)	<.001
II	189 (0.9)	31 (1.0)	158 (0.8)	
III	3324 (15.0)	393 (12.5)	2931 (15.4)	
IV	18 686 (84.1)	2730 (86.5)	15 956 (83.7)	
Etiology of heart failure				
Congenital heart disease	114 (0.5)	6 (0.2)	108 (0.6)	<.001
Ischemic	9749 (43.9)	1520 (48.2)	8229 (43.2)	
NICM	11 717 (52.7)	1550 (49.1)	10 167 (53.3)	
Restrictive CM	444 (2.0)	54 (1.7)	390 (2.0)	
Unknown	197 (0.9)	26 (0.8)	171 (0.9)	
Admitting diagnosis or planned implant				
Heart failure	14 645 (65.9)	1727 (54.7)	12 918 (67.8)	<.001
Cardiac surgery	261 (1.2)	65 (2.1)	196 (1.0)	
Noncardiac medical problem	160 (0.7)	36 (1.1)	124 (0.7)	
LVAD placement	5861 (26.4)	1124 (35.6)	4737 (24.8)	
Total artificial heart placement	3 (0.0)	0	3 (0.0)	
Other cardiology	661 (3.0)	101 (3.2)	560 (2.9)	
Acute MI	571 (2.6)	98 (3.1)	473 (2.5)	
Noncardiac surgery	11 (0.0)	3 (0.1)	8 (0.0)	
Unknown	48 (0.2)	2 (0.1)	46 (0.2)	
ICD	17 508 (78.8)	2542 (80.5)	14 966 (78.5)	.01
Comorbidities				
Any transplant-limiting comorbidity	13 456 (60.6)	2435 (77.2)	11 021 (57.8)	<.001
Chronic kidney disease	1584 (7.1)	293 (9.3)	1291 (6.8)	<.001
Current tobacco use	1146 (5.2)	220 (7.0)	926 (4.9)	<.001
Frailty	1098 (4.9)	261 (8.3)	837 (4.4)	<.001
History of tobacco use	1748 (7.9)	325 (10.3)	1423 (7.5)	<.001
Obesity	2675 (12.0)	466 (14.8)	2209 (11.6)	<.001
Limited social support	873 (3.9)	123 (3.9)	750 (3.9)	.96
Implant year				
2012	1639 (7.4)	177 (5.6)	1462 (7.7)	<.001
2013	2557 (11.5)	281 (8.9)	2276 (11.9)	
2014	2678 (12.1)	297 (9.4)	2381 (12.5)	
2015	2993 (13.5)	382 (12.1)	2611 (13.7)	
2016	2688 (12.1)	321 (10.2)	2367 (12.4)	
2017	2461 (11.1)	402 (12.7)	2059 (10.8)	
2018	2864 (12.9)	500 (15.8)	2364 (12.4)	
2019	3083 (13.9)	570 (18.1)	2513 (13.2)	
2020	1258 (5.7)	226 (7.2)	1032 (5.4)	

Abbreviations: BMI, body mass index (calculated as weight in kilograms divided by height in meters squared); CM, cardiomyopathy; HMO, health maintenance organization; ICD, implantable cardioverter-defibrillator; LVAD, left ventricular assist device; MI, myocardial infarction; NICM, nonischemic cardiomyopathy; NYHA, New York Heart Association; STS, Society of Thoracic Surgeons.

^a^ *P* values determined using 2-sided Mann-Whitney test for continuous variables and χ^2^ tests for categorical variables.

^b^ Race was reported by sites and categorized as American Indian or Alaska Native, Asian, African American or Black, Hawaiian or other Pacific Islander, White, Unknown/Undisclosed, or Other/none of the above. Due to low numbers in groups, race was categorized as White, Black, or other in this analysis.

A BTT strategy was used for 37.7% (n = 7186) of patients who received implants at LVAD/transplant centers and 16.9% (n = 532) of those who received implants at LVAD-only centers. Results of the multivariable logistic regression exploring the association between transplant center status and BTT listing are displayed in Table 2. Compared with LVAD-only centers, there was a 1.79 (95% CI, 1.35-2.38; *P* < .001) increased odds of a patient being considered for transplant (implant as BTT LVAD) at LVAD/transplant centers. Patients presenting to an LVAD-only center were a mean of 6.21% (95% CI, 3.27%-9.15%) less likely to receive an LVAD with a BTT intent (posterior estimation, 0.31; 95% CI, 0.28-0.33) than patients at an LVAD/transplant center (posterior estimation, 0.37; 95% CI, 0.35-0.39) after adjusting for patient clinical and center characteristics. In the sensitivity analysis evaluating a transplant volume association (eTable 4 in Supplement 1), there was a significant increase in the odds of BTT listing at LVAD/transplant centers that performed fewer than 10 transplants annually compared with LVAD-only centers (odds ratio, 1.48; 95% CI, 1.05-2.08). There was a significant trend in increased odds of BTT listing among centers with increasing transplant volume (*P* value trend test <.001). Among centers performing more than 60 transplants in the year of LVAD implant, there was a 2.77 (95% CI, 1.87-4.09) increased odds of implanting an LVAD in a patient for a BTT strategy vs LVAD-only centers.

**Table 2.** Association of Transplant Center Status With Bridge-to-Transplant Listing Strategy

Variable	OR (95% CI)	*P* value
Type of center		
LVAD only	1 [Reference]	NA
LVAD/transplant	1.79 (1.35-2.38)	<.001
Device type		
Axial	1 [Reference]	NA
Hybrid magnetically levitated	8.21 (7.23-9.31)	<.001
Fully magnetically levitated	13.60 (11.20-16.51)	<.001
Age (per year)	0.96 (0.95-0.96)	<.001
BMI	0.98 (0.98-0.99)	<.001
Albumin (per 1 g/dL)	1.25 (1.17-1.35)	<.001
Total bilirubin (per mg/dL)	0.93 (0.90-0.95)	<.001
Right atrial pressure (per 1 mm Hg)	0.99 (0.99-1.00)	.01
Race^a^		
Black	0.84 (0.76-0.94)	.002
White	1 [Reference]	NA
Other	0.78 (0.67-0.91)	.002
Presence of ICD		
No	1 [Reference]	NA
Yes	1.35 (1.22-1.50)	<.001
NYHA functional class		
IV	1 [Reference]	NA
I-III	1.25 (1.11-1.40)	<.001
Educational level		
Less than high school	1 [Reference]	NA
High school	1.12 (1.00-1.27)	.06
Above high school	1.39 (1.23-1.56)	<.001
Marital status		
Single	1 [Reference]	NA
Married	1.61 (1.43-1.80)	<.001
Divorced/separated or other	1.44 (1.26-1.65)	<.001
Payer		
Medicare	1 [Reference]	NA
Medicaid	0.95 (0.78-1.16)	.61
Commercial and HMO	1.29 (1.11-1.49)	<.001
Other	1.31 (1.11-1.54)	.002
Primary diagnosis		
Ischemic CM	1 [Reference]	NA
Congenital heart disease	1.04 (0.60-1.82)	.88
NICM	1.24 (1.12-1.37)	<.001
Restrictive CM	0.87 (0.64-1.17)	.34
Unknown	0.65 (0.42-0.99)	.05
Implant year		
2012	1 [Reference]	NA
2013	0.59 (0.49-0.70)	<.001
2014	0.51 (0.42-0.61)	<.001
2015	0.43 (0.40-0.52)	<.001
2016	0.36 (0.30-0.44)	<.001
2017	0.24 (0.19-0.30)	<.001
2018	0.08 (0.06-0.10)	<.001
2019	0.03 (0.02-0.03)	<.001
2020	0.02 (0.02-0.03)	<.001
UNOS region		
1	1 [Reference]	NA
2	0.49 (0.25-0.98)	.04
3	0.30 (0.15-0.62)	.001
4	0.41 (0.18-0.90)	.03
5	0.60 (0.30-1.20)	.14
6	0.81 (0.31-2.14)	.67
7	0.50 (0.24-1.05)	.07
8	0.79 (0.33-1.88)	.59
9	0.82 (0.38-1.76)	.60
10	0.46 (0.21-0.97)	.04
11	0.46 (0.22-0.94)	.03
No. of hospital beds		
≥500	1 [Reference]	NA
50-99	0.94 (0.22-4.05)	.93
100-199	0.96 (0.17-5.43)	.96
200-299	1.53 (0.77-3.03)	.22
300-399	1.86 (1.05-3.30)	.03
400-499	0.90 (0.58-1.37)	.61
ACGME programs		
Yes	1 [Reference]	NA
No	0.43 (0.24-0.78)	.006
Hospital authority		
State run	1 [Reference]	NA
Hospital district	0.86 (0.41-1.79)	.68
Not-for-profit church	0.87 (0.46-1.66)	.68
Other not for profit	0.79 (0.50-1.26)	.33
For-profit partnership	1.01 (0.25-4.04)	.99
For-profit corporation	0.65 (0.26-1.62)	.35
Transplant-limiting comorbidity		
None	1 [Reference]	NA
Any	0.05 (0.05-0.05)	<.001
Prior CABG	0.79 (0.70-0.90)	<.001

Abbreviations: ACGME, Accreditation Council for Graduate Medical Education; BMI, body mass index; CABG, coronary artery bypass graft surgery; CM, cardiomyopathy; HMO, health maintenance organization; ICD, implantable cardioverter-defibrillator; LVAD, left ventricular assist device; NA, not applicable; NICM, nonischemic cardiomyopathy; NYHA, New York Heart Association; OR, odds ratio; UNOS, United Network for Organ Sharing.

^a^ Race was reported by sites and categorized as American Indian or Alaska Native, Asian, African American or Black, Hawaiian or other Pacific Islander, White, Unknown/Undisclosed, or Other/none of the above. Due to low numbers in groups, race was categorized as White, Black, or other in this analysis.

**Figure 2.**
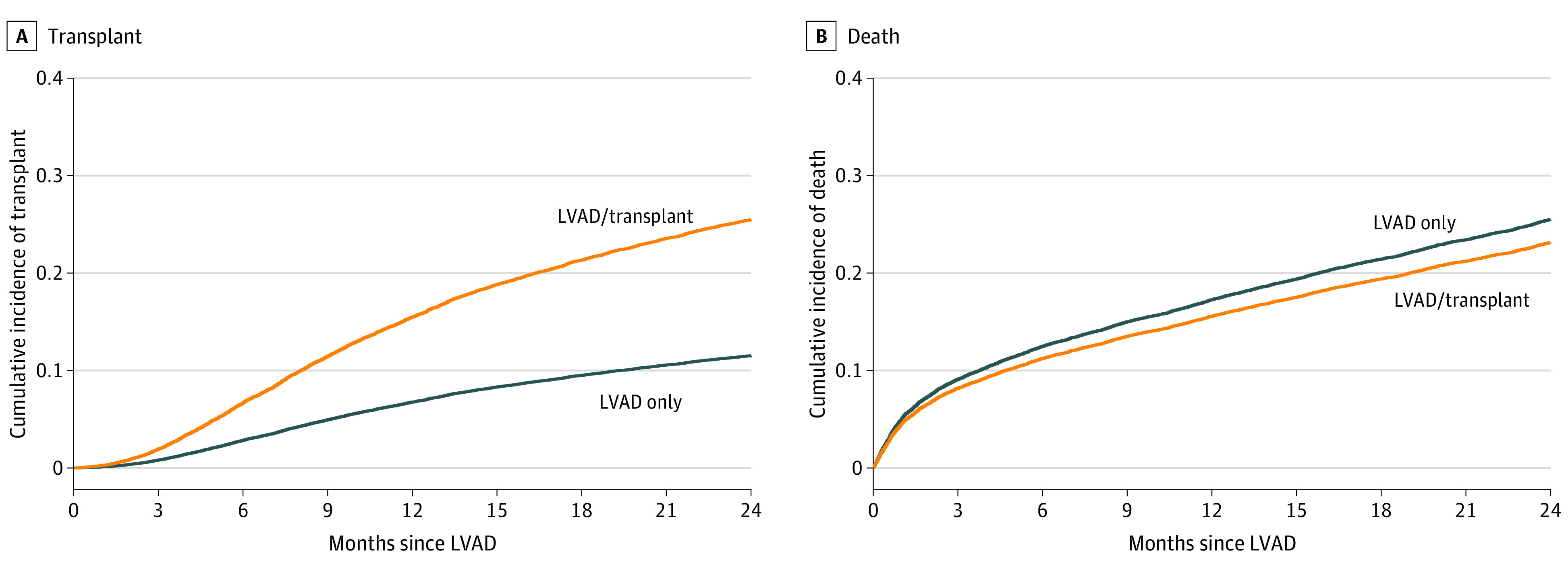
Cumulative Incidence of Transplant and Death in the 2 Years Following Left Ventricular Assist Device (LVAD) Implant by Center Status A, The rate of heart transplant in the 2 years following LVAD implant was 25.6% at LVAD/transplant centers and 11.9% at LVAD-only centers (*P* < .001). B, There was no significant difference in the rate of death at LVAD/transplant centers (23.9%) and LVAD-only centers (26.4%) (*P* = .66). The number at risk is not reported given the presence of a competing risk.^18^

The 2-year transplant rate following LVAD implant was 25.6% at LVAD/transplant centers and 11.9% at LVAD-only centers; death occurred in 23.9% of patients at LVAD/transplant centers and 26.4% at LVAD-only centers (Figure 2^18^). The multivariable cause-specific Cox proportional hazard model results are shown in Table 3. In LVAD/transplant centers, LVAD recipients had an associated 33% increased rate of transplant compared with LVAD-only centers at any given time during 2 years (adjusted hazard ratio [HR], 1.33; 95% CI, 1.17-1.51). Rates of death while receiving LVAD support were equivalent at 2 years between center types in the multivariable model (adjusted HR, 0.99; 95% CI, 0.90-1.08) (eTable 5 in Supplement 1).

**Table 3.** Results of Multivariable Cause-Specific Cox Proportional Hazards Regression Model for Transplant in the 2 Years Following LVAD

Variable	HR (95% CI)	*P* value
Type of center		
LVAD only	1 [Reference]	NA
LVAD/transplant	1.33 (1.17-1.51)	<.001
Device type		
Axial	1 [Reference]	NA
Hybrid magnetically levitated	1.57 (1.46-1.70)	<.001
Fully magnetically levitated	1.20 (1.04-1.38)	.01
Age (per year)	0.99 (0.99-0.99)	<.001
Blood type		
O	1 [Reference]	NA
A	1.63 (1.53-1.74)	<.001
B	1.67 (1.52-1.82)	.65
AB	2.31 (1.99-2.67)	<.001
BMI	0.98 (0.97-0.98)	<.001
Right atrial pressure, per 1 mm Hg	1.01 (1.00-1.01)	.001
Race^a^		
Black	0.73 (0.68-0.79)	<.001
White	1 [Reference]	NA
Other	0.85 (0.77-0.94)	.002
Presence of ICD		
No	1 [Reference]	NA
Yes	1.20 (1.12-1.30)	<.001
Ventilator use during hospitalization	1.27 (1.12-1.43)	<.001
Marital status		
Single	1 [Reference]	NA
Married	1.21 (1.11-1.32)	.32
Divorced/separated or other	1.16 (1.05-1.28)	.004
Payer		
Medicare	1 [Reference]	NA
Medicaid	0.97 (0.82-1.16)	.74
Commercial and HMO	1.62 (1.44-1.82)	<.001
Other	1.29 (1.14-1.46)	<.001
Primary diagnosis		
Ischemic CM	1 [Reference]	NA
Congenital heart disease	0.75 (0.50-1.12)	.16
NICM	1.13 (1.05-1.22)	.001
Restrictive CM	0.84 (0.67-1.06)	.14
Unknown	0.77 (0.54-1.08)	.13
Implant year		
2012	1 [Reference]	NA
2013	0.78 (0.68-0.89)	<.001
2014	0.75 (0.66-0.85)	<.001
2015	0.83 (0.73-0.94)	.004
2016	0.89 (0.78-1.02)	.09
2017	0.77 (0.66-0.91)	.002
2018	0.66 (0.56-0.79)	<.001
2019	0.47 (0.38-0.58)	<.001
2020	0.38 (0.25-0.57)	<.001
UNOS region		
1	1 [Reference]	NA
2	0.64 (0.54-0.75)	<.001
3	0.54 (0.46-0.65)	<.001
4	0.52 (0.44-0.62)	<.001
5	0.82 (0.70-0.95)	<.001
6	0.93 (0.76-1.14)	<.001
7	0.66 (0.56-0.77)	.008
8	0.78 (0.64-0.95)	.49
9	0.69 (0.58-0.83)	<.001
10	0.64 (0.55-0.75)	.01
11	0.64 (0.55-0.76)	<.001
No. of hospital beds		
≥500	1 [Reference]	NA
50-99	2.59 (1.14-5.88)	.02
100-199	1.94 (0.86-4.38)	.11
200-299	0.93 (0.80-1.09)	.37
300-399	1.66 (1.39-1.98)	<.001
400-499	1.45 (1.32-1.58)	<.001
US city rank size		
1-25	1 [Reference]	NA
26-50	0.74 (0.66-0.82)	<.001
51-75	0.89 (0.78-1.00)	.06
76-100	1.00 (0.87-1.16)	.95
Greater than top 100	0.63 (0.58-0.69)	<.001
Medical school affiliation		
Yes	1 [Reference]	NA
No	0.26 (0.18-0.38)	<.001
Hospital authority		
State run	1 [Reference]	NA
Hospital district	1.04 (0.89-1.22)	.61
Not-for-profit church	0.56 (0.48-0.66)	<.001
Other not for profit	0.82 (0.75-0.91)	<.001
For-profit partnership	1.01 (0.70-1.46)	.95
For-profit corporation	0.52 (0.38-0.71)	<.001
Transplant-limiting comorbidity		
None	1 [Reference]	NA
Any	0.36 (0.34-0.39)	<.001
Prior CABG	0.83 (0.75-0.92)	<.001

Abbreviations: BMI, body mass index; CABG, coronary artery bypass graft surgery; CM, cardiomyopathy; HMO, health maintenance organization; HR, hazard ratio; ICD, implantable cardioverter-defibrillator; LVAD, left ventricular assist device; NA, not applicable; NICM, nonischemic cardiomyopathy; UNOS, United Network for Organ Sharing.

^a^ Race was reported by sites and categorized as American Indian or Alaska Native, Asian, African American or Black, Hawaiian or other Pacific Islander, White, Unknown/Undisclosed, or Other/none of the above. Due to low numbers in groups, race was categorized as White, Black, or other in this analysis.

### Robustness and Sensitivity Analyses

There was a significant interaction between center type and device strategy (eTable 6 in Supplement 1). Compared with patients at LVAD-only centers, patients receiving implants at LVAD/transplant centers had associated increased transplant rates of 25% for BTT (HR, 1.25; 95% CI, 1.05-1.47) and 63% (HR, 1.63; 95% CI, 1.31-2.04) for destination therapy; bridge to candidacy had equivalent rates (HR, 1.05; 95% CI, 0.79-1.40). The inclusion of device strategy in the model (eTable 7 in Supplement 1) and limiting the sample to 19 143 recipients younger than 70 years (eTable 8 in Supplement 1) did not appreciably change the findings between center status and outcomes. Among patients younger than 70 years, those receiving an LVAD at an LVAD/transplant center had a 29% higher transplant rate (HR, 1.29; 95% CI, 1.11-1.46). Not including right atrial pressure as a candidate variable (eTable 9 in Supplement 1) and using a cutoff of *P* < .10 to select variables in the multivariable model were not associated with changes in the results (eTable 10 and eTable 11 in Supplement 1). Results of the propensity score–matched analysis were similar. Among the 1:1 matched 6266 LVAD recipients, transplant occurred in 16.2% (n = 509) of patients at LVAD/transplant centers and 10.0% (n = 310) of patients at LVAD-only centers; death occurred in 22.7% (n = 713) of patients at LVAD/transplant centers and 22.6% (n = 709) of patients at LVAD-only centers. In adjusted models, there was an associated 35% increased rate (HR, 1.35; 95% CI, 1.16-1.59) of transplant at LVAD/transplant centers (eTable 12 in Supplement 1).

## Discussion

This contemporary national analysis of durable LVAD implant documents systemic inequities in transplant access for patients receiving a durable LVAD at LVAD-only centers. Receiving a durable LVAD at a center that also performs heart transplants was associated with a higher odds of patients being considered a transplant candidate at the time of LVAD implant and with increased rates of heart transplant in the 2 years following LVAD implant. There was no significant difference in postimplant LVAD mortality between LVAD/transplant and LVAD-only centers within 2 years of follow-up.

A 2018 study reported similar 1-year outcomes for death and major adverse events for patients receiving durable LVAD therapy at transplant or nontransplant LVAD centers.^19^ The present study builds on these findings by noting no significant differences in LVAD survival up to 2 years following LVAD implant in this contemporary cohort.

To our knowledge, the finding of an associated increase in heart transplant after LVAD at centers that perform transplants is novel. Nontransplant LVAD centers are increasing in number. The CMS national coverage determination has made establishing an LVAD-only program less restrictive now that there is no longer the need to form a relationship with a Medicare-approved heart transplant center.^9^ The decreased barriers to establishing a durable LVAD program at a non–Medicare-approved heart transplant center will likely lead to continued increases in the number of nontransplant centers and LVAD volumes at these centers—trends that were already occurring before the change. Although there should be enthusiasm for the potential of LVAD-only centers to increase access to LVAD, it appears that receiving an LVAD at a center that does not perform transplants results in differential assessment of transplant eligibility at the time of LVAD implant and inequities in receipt of transplant. This adverse association between transplant eligibility and access for patients receiving an LVAD at an LVAD-only center occurred before the CMS policy change that removed the requirement for shared care for LVAD candidates being considered for transplant at a Medicare-approved transplant center. This finding is important because the lower levels of transplant eligibility and access may worsen under the new, less restrictive CMS policy and heart transplant remains the best long-term treatment strategy for most eligible patients with advanced HF because long-term survival outcomes are dramatically different.^10,20,21^

In addition, the finding of the interaction of therapeutic intent and receipt of an LVAD merits discussion. Recent work from the Multicenter Study of MagLev Technology in Patients Undergoing Mechanical Circulatory Support Therapy With HeartMate 3 trial has documented that LVADs effectively improve quality of life and survival regardless of therapeutic intent.^22^ With this result, and recognizing the challenges in identifying LVAD intent and dynamic changes in patient status,^23,24^ CMS removed the therapeutic intent designation.^9^ In the present study, there was an association between having a transplant program and differential determination of transplant candidacy and increased access to transplant after LVAD implant, not only for patients initially considered transplant candidates (BTT) but also for destination therapy intent patients at LVAD/transplant centers. This finding could occur because of disparities in either reevaluation for transplant or improvements in transplant candidacy after LVAD for patients at LVAD/transplant centers (eg, improved kidney function following LVAD implant).^25^ Determining transplant eligibility is complex,^26^ particularly given the dynamic course of HF.^23,24^ A patient with advanced HF may be ineligible because of comorbidities, substance use, psychological concerns, or inadequate social support.^26,27^ LVAD/transplant centers appear to be more aggressively pursuing transplants even for patients initially considered ineligible.

These collective results may raise questions for CMS policy makers, particularly when considering the modifications to the adult heart allocation system.^28^ The US adult heart allocation system was modified by the Organ Procurement and Transplantation Network in October 2018 as part of a strategic goal to provide equity in access to transplant by prioritizing those at the highest risk for death on the waiting list. The revisions to the Organ Procurement and Transplantation Network/United Network for Organ Sharing adult heart allocation system have resulted in widespread changes in treatment strategies for patients being considered for transplant, with significantly decreased use of durable LVADs as a bridging strategy and a corresponding increased use of short-term mechanical circulatory support devices to bridge patients to transplant, which itself appears to have reduced equity in transplant.^28,29,30^ The changes have significantly reduced the likelihood of transplant after durable LVAD implant unless candidates are listed at higher urgency status due to an LVAD complication or clinical deterioration.^30^ The reality is that durable LVADs are much less likely to be a bridge to the best therapy (ie, transplant) in the current allocation system. As a result, there is a critical need to select appropriate durable LVAD and transplant candidates at the initial evaluation. As currently designed and implemented, the CMS policy will likely further challenge equity in access to transplant for patients seeking care at nontransplant centers and may have the unintended consequence of contributing to increasing inequities in access to transplants, as has been feared.^24^

There is a potential role of registries to monitor equity in access to advanced therapies, although there is not currently a registry that includes candidates for both LVAD and transplant. Rather, only patients who receive an LVAD (STS Intermacs) or are listed for transplant (United Network for Organ Sharing) are included in a registry. Although we agree with the views expressed in the recent letter endorsed by the STS, the American College of Cardiology, the Heart Failure Society of America, and the American Association for Thoracic Surgery about the opportunity to use registry data to compare practices between institutions to ensure appropriate consideration before LVAD,^31^ our work suggests there is a need to ensure most patients without an obvious contraindication to transplant (eg, active tobacco use) are evaluated at transplant centers before durable LVAD and following LVAD implant to assess ongoing transplant eligibility. In addition to monitoring access to transplants, determining whether the increased access will impact documented inequities in access to LVAD among underserved groups, including women and Black patients with HF, will remain critical.^32,33,34,35^

### Limitations

This study has several limitations. The study used observational registry data, which likely have unobserved heterogeneity, unmeasured confounding, and possible underreporting of outcomes data despite routine hospital audits by STS Intermacs and the low level of overall missingness for candidate variables. Nonetheless, STS Intermacs remains the most comprehensive registry of durable mechanical circulatory support devices with detailed patient-level variables in the US and is well positioned to support evaluations of variation in practice and outcomes. Furthermore, similar results using multiple rigorous sensitivity analyses, including propensity score–matching methods, support the robustness of the study findings. Patients undergoing transplants were censored during LVAD survival analyses, and information on urgent transplant listing for device complications was not available. In addition, this analysis focused on outcomes at 2 years. Thus, this study cannot examine whether longer-term outcomes may be different between center types.

## Conclusions

The findings of this study suggest systemically different approaches to consideration for transplant candidacy at LVAD centers that perform transplants compared with LVAD-only centers. Furthermore, LVAD recipients at nontransplant centers may have reduced access to transplants. Recent CMS policy changes will likely have the unintended consequence of increasing disparities in access to transplants despite increasing LVAD access.

## Notes

Supplement 1. **eTable 1.** Candidate Risk Factors for Multivariable Modeling **eTable 2.** Variables Used for Propensity Matching **eTable 3.** Center Characteristics of LVAD Recipients **eTable 4.** Results of Multivariable Adjusted Models for the Interaction Between Transplant Center Status and BTT Listing Strategy **eTable 5.** Results of Multivariable Adjusted Survival Models for Mortality With Transplant as a Competing Risk **eTable 6.** Results of Multivariable Adjusted Survival Models for the Interaction Between Transplant Center Status and Listing Strategy and Transplant **eTable 7.** Results of Multivariable Adjusted Survival Models With Inclusion of Device Strategy **eTable 8.** Results of Multivariable Adjusted Survival Models Limited to Patients Under the Age of 70 Years **eTable 9.** Results of Multivariable Adjusted Survival Models Not Including Right Atrial Pressure in the Multivariable Model **eTable 10.** Association of Transplant Center Status With BTT-Listing Strategy Using a *P* Value Cutoff of <.10 to Select Variables in the Final Multivariable Model **eTable 11.** Results of Multivariable Cause-Specific Cox Model for Transplant in the 2 Years Following LVAD Using a *P* Value Cutoff of <.10 to Select Variables in the Final Multivariable Model **eTable 12.** Results of Multivariable Cause-Specific Cox Model for Transplant in the 2 Years Following LVAD Among 6266 Propensity Matched Recipients **eFigure.** Construction of Cohort **eMethods 1.** Additional Information on The Society of Thoracic Surgeons (STS) Intermacs Database **eMethods 2.** Determination of Therapeutic Intent Categorization
eReferences

Supplement 2. **Nonauthor Collaborators.** Michigan Congestive Heart Failure Investigators
