# Supplementary material for: Comparison of Evaluations for Heart Transplant Before Durable Left Ventricular Assist Device and Subsequent Receipt of Transplant at Transplant vs Nontransplant Centers
Source: JAMA Netw Open. 2022 Nov 7;5(11):e2240646. doi: 10.1001/jamanetworkopen.2022.40646 (PMC9641540; doi:10.1001/jamanetworkopen.2022.40646)
Supplement: Supplement 1. — eTable 1. Candidate Risk Factors for Multivariable Modeling eTable 2. Variables Used for Propensity Matching eTable 3. Center Characteristics of LVAD Recipients eTable 4. Results of Multivariable Adjusted Models for the Interaction Between Transplant Center Status and BTT Listing Strategy eTable 5. Results of Multivariable Adjusted Survival Models for Mortality With Transplant as a Competing Risk eTable 6. Results of Multivariable Adjusted Survival Models for the Interaction Between Transplant Center Status and Listing Strategy and Transplant eTable 7. Results of Multivariable Adjusted Survival Models With Inclusion of Device Strategy eTable 8. Results of Multivariable Adjusted Survival Models Limited to Patients Under the Age of 70 Years eTable 9. Results of Multivariable Adjusted Survival Models Not Including Right Atrial Pressure in the Multivariable Model eTable 10. Association of Transplant Center Status With BTT-Listing Strategy Using a P Value Cutoff of <.10 to Select Variables in the Final Multivariable Model eTable 11. Results of Multivariable Cause-Specific Cox Model for Transplant in the 2 Years Following LVAD Using a P Value Cutoff of <.10 to Select Variables in the Final Multivariable Model eTable 12. Results of Multivariable Cause-Specific Cox Model for Transplant in the 2 Years Following LVAD Among 6266 Propensity Matched Recipients eFigure. Construction of Cohort eMethods 1. Additional Information on The Society of Thoracic Surgeons (STS) Intermacs Database eMethods 2. Determination of Therapeutic Intent Categorization eReferences [file jamanetwopen-e2240646-s001.pdf]

Supplementary Online Content

Cascino TM, McCullough JS, Wu X, et al; Michigan Congestive Heart Failure Investigators. Comparison of evaluations for heart transplant before durable left ventricular assist device and subsequent receipt of transplant at transplant vs nontransplant centers. *JAMA Netw Open*. 2022;5(11):e2240646. doi:10.1001/jamanetworkopen.2022.40646

eTable 1. Candidate Risk Factors for Multivariable Modeling

eTable 2. Variables Used for Propensity Matching

eTable 3. Center Characteristics of LVAD Recipients

eTable 4. Results of Multivariable Adjusted Models for the Interaction Between Transplant Center Status and BTT Listing Strategy

eTable 5. Results of Multivariable Adjusted Survival Models for Mortality With Transplant as a Competing Risk

eTable 6. Results of Multivariable Adjusted Survival Models for the Interaction Between Transplant Center Status and Listing Strategy and Transplant

eTable 7. Results of Multivariable Adjusted Survival Models With Inclusion of Device Strategy

eTable 8. Results of Multivariable Adjusted Survival Models Limited to Patients Under the Age of 70 Years

eTable 9. Results of Multivariable Adjusted Survival Models Not Including Right Atrial Pressure in the Multivariable Model

eTable 10. Association of Transplant Center Status With BTT-Listing Strategy Using a *P* Value Cutoff of <.10 to Select Variables in the Final Multivariable Model

eTable 11. Results of Multivariable Cause-Specific Cox Model for Transplant in the 2 Years Following LVAD Using a *P* Value Cutoff of <.10 to Select Variables in the Final Multivariable Model

eTable 12. Results of Multivariable Cause-Specific Cox Model for Transplant in the 2 Years Following LVAD Among 6266 Propensity Matched Recipients

eFigure. Construction of Cohort

eMethods 1. Additional Information on The Society of Thoracic Surgeons (STS) Intermacs Database

eMethods 2. Determination of Therapeutic Intent Categorization

eReferences

This supplementary material has been provided by the authors to give readers additional information about their work.

eTable 1. Candidate Risk Factors for Multivariable Modeling

Variable Name	Original Location	Label
ADMISSION_REASON	STS Intermacs	Admitting diagnosis or planned implant
AGE	STS Intermacs	Age at implant
ALBUMIN_G_DL	STS Intermacs	Albumin (g/dL)
AORTIC_REGURG	STS Intermacs	Aortic regurgitation
BILI_TOTAL_MG_DL	STS Intermacs	Total bilirubin (mg/dL)
BLOOD_TYPE	STS Intermacs	Blood type
BMI	STS Intermacs	Body mass index(kg/m2)
BSA	STS Intermacs	Body surface area(m2)
BUN_MG_DL	STS Intermacs	Blood urea nitrogen(mg/dL)
CARDIAC_INDEX	STS Intermacs	Cardiac index ($l/min/m^2$)
CC2_Combined		Concern or contradiction that is a limitation for transplant listing (advanced age or allosensitization or chronic coagulopathy or chronic infection concerns or renal disease or contraindication to immunosuppression or current smoking or frailty or frequent ICD shocks or heparin induced thrombocytopenia or history of alcohol abuse or history of atrial arrhythmia or history of bone marrow transplant or history of drug use or history of HIV or history of lymphoma/leukemia or history of smoking or history of solid organ cancer or history of GI ulcers or history of hepatitis or large BMI or limited social support or liver dysfunction or limited cognitive understanding or major stroke or malnutrition/cachexia or musculoskeletal limitation or narcotic dependence or other cerebrovascular disease or other major psychological diagnosis or other comorbidity or peripheral vascular disease or pulmonary hypertension or patient does not want a transplant or recent pulmonary embolus or non-compliance or severe depression or severe diabetes or thoracic aortic disease or unfavorable mediastinal anatomy)
CREAT_MG_DL	STS Intermacs	Creatinine (mg/dL)
CURRENT_ICD	STS Intermacs	Current implantable cardioverter-defibrillator

© 2022 Cascino TM et al. *JAMA Network Open.*

DEVICE_STRATEGY	STS Intermacs	Current device strategy (BTT, BTD, or DT)
device_type	STS Intermacs	Device type (axial, hybrid magnetically levitated or fully magnetically levitated)
DIA_BP	STS Intermacs	Diastolic blood pressure (mmHg)
EDUC_LEVEL	STS Intermacs	Education level
ETHNICITY	STS Intermacs	Ethnicity
EVENT_HOSP_COMBINE_SURG	STS Intermacs	Events this Hospitalization (aneurysmectomy or aortic valve replacement/repair or coronary artery bypass grafting or cardiac surgery, other or mitral valve replacement/repair)
EVENT_HOSP_CAR_ARREST	STS Intermacs	Events this Hospitalization (cardiac arrest)
EVENT_HOSP_CAR_SUR_OTHER	STS Intermacs	Events this hospitalization (other cardiac surgery)
EVENT_HOSP_CON_CAR_SUR	STS Intermacs	Events this hospitalization (congenital cardiac surgery)
EVENT_HOSP_DIALYSIS	STS Intermacs	Events this hospitalization (dialysis)
EVENT_HOSP_ECMO	STS Intermacs	Events this hospitalization (extracorporeal membrane oxygenation)
EVENT_HOSP_FEED_TUBE	STS Intermacs	Events this hospitalization (feeding tube)
EVENT_HOSP_IABP	STS Intermacs	Events this hospitalization (intra-aortic balloon pump)
EVENT_HOSP_INTUB_VENTILAT	STS Intermacs	Events this hospitalization (intubation)
EVENT_HOSP_MAJOR_INFEC	STS Intermacs	Events this hospitalization (major infections)
EVENT_HOSP_MAJOR_MI	STS Intermacs	Events this hospitalization (major myocardial infarction)
EVENT_HOSP_NONE	STS Intermacs	Events this hospitalization (none)

© 2022 Cascino TM et al. *JAMA Network Open.*

EVENT_HOSP_POS_BLD_CULT	STS Intermacs	Events this hospitalization (positive blood culture)
EVENT_HOSP_ULTRAFILT	STS Intermacs	Events this hospitalization (ultrafiltration)
EVENT_HOSP_VENTILAT	STS Intermacs	Events this hospitalization (ventilator prior to LVAD)
Gender	STS Intermacs	Male or female
IMPL_YR	STS Intermacs	Implant year
HEMOGLOBIN_G_DL	STS Intermacs	Hemoglobin (g/dL)
HEMO_A1C_PERCENT	STS Intermacs	Hemoglobin A1C (percent)
HGT_CM	STS Intermacs	Height (cm)
HOSP_CTRL_UNOHOSP_INDX _UNOS_REGIONS_REGION	STS Intermacs	UNOS region
HOSPITAL_ID_INDEX_DEIDENT	STS Intermacs	STS-Intermacs institution identifier
HR_RATE	STS Intermacs	Heart rate
INR	STS Intermacs	International normalized ratio
INTERVENTION_48_HRS_ANEURS	STS Intermacs	Intervention within last 48 hours (aneurysmectomy)
INTERVENTION_48_HRS_AVR	STS Intermacs	Intervention within last 48 hours (aortic valve replacement/repair)
INTERVENTION_48_HRS_CABG	STS Intermacs	Intervention within last 48 hours (coronary artery bypass grafting)
INTERVENTION_48_HRS _CON_CAR_SUR	STS Intermacs	Intervention within last 48 hours (congenital cardiac surgery)
INTERVENTION_48_HRS_DIALY	STS Intermacs	Intervention within last 48 hours (dialysis)
INTERVENTION_48_HRS_ECMO	STS Intermacs	Intervention within last 48 hours (extracorporeal membrane oxygenation)
INTERVENTION_48 _HRS_FEED_TUBE	STS Intermacs	Intervention within last 48 hours (feeding tube)
INTERVENTION_48_HRS_IABP	STS Intermacs	Intervention within last 48 hours (intra-aortic balloon pump)
INTERVENTION_48_HRS_MVR	STS Intermacs	Intervention within last 48 hours (mitral valve replacement/repair)
INTERVENTION_48_HRS_NONE	STS Intermacs	Intervention within last 48 hours (none)

© 2022 Cascino TM et al. *JAMA Network Open.*

INTERVENTION_48_HRS_RVAD	STS Intermacs	Intervention within last 48 hours (RVAD)
INTERVENTION_48_HRS_TAH	STS Intermacs	Intervention within last 48 hours (TAH)
INTERVENTION_48_HRS_ULTRAFILT	STS Intermacs	Intervention within last 48 hours (ultrafiltration)
INTERVENTION_48_HRS_VENTILAT	STS Intermacs	Intervention within last 48 hours (ventilator)
IV_INO_THERAPY_AGENTS_COMBINE	STS Intermacs	Intravenous inotrope therapy agents (Dobutamine or Levsimendan or Milrinone)
IV_INO_THERAPY_AGENTS_DOPA	STS Intermacs	Intravenous inotrope therapy agents (Dopamine)
IV_INO_THERAPY_AGENTS_EPINEPH	STS Intermacs	Intravenous inotrope therapy agents (Epinephrine)
IV_INO_THERAPY_AGENTS_ISOPRO	STS Intermacs	Intravenous inotrope therapy agents (Isoproteronol)
IV_INO_THERAPY_AGENTS_NOREPI	STS Intermacs	Intravenous inotrope therapy agents (Norepinephrine)
MARITAL_STAT	STS Intermacs	Marital status
NYHA	STS Intermacs	New York Heart Association Class
PLATELET_X10_3_UL	STS Intermacs	Platelets (x10/uL)
POTASSIUM_MEQ_L	STS Intermacs	Potassium (mEq/L)
RA_PRES	STS Intermacs	Right atrial pressure
CV_PRES	STS Intermacs	Central venous pressure
Payor	STS Intermacs	Health insurance
PREV_CARD_OP_CON_SUR_AP_SH	STS Intermacs	Previous cardiac operation (AP shunt)
PREV_CARD_OP_CON_SUR_ART_SWCH	STS Intermacs	Previous cardiac operation (arterial switch)
PREV_CARD_OP_CON_SUR_ASD	STS Intermacs	Previous cardiac operation (atrial septal defect repair)

© 2022 Cascino TM et al. *JAMA Network Open.*

PREV_CARD_OP_CON_SUR_CLASSIC	STS Intermacs	Previous cardiac operation (Congenitally Corrected Transposition Repair (classic))
PREV_CARD_OP_CON_SUR_DBL_SWCH	STS Intermacs	Previous cardiac operation (Congenitally Corrected Transposition Repair (double switch))
PREV_CARD_OP_CON_SUR_DKS	STS Intermacs	Previous cardiac operation (Damus Kaye Stansel)
PREV_CARD_OP_CON_SUR_EBSTEIN	STS Intermacs	Previous cardiac operation (Ebstein's Anomaly Repair)
PREV_CARD_OP_CON_SUR_FONTAN	STS Intermacs	Previous cardiac operation (Fontan Procedure)
PREV_CARD_OP_CON_SUR_GLN_BI	STS Intermacs	Previous cardiac operation (Glenn, Bi-directional)
PREV_CARD_OP_CON_SUR_GLN_CL	STS Intermacs	Previous cardiac operation (Glenn, Classical)
PREV_CARD_OP_CON_SUR_NORWOOD	STS Intermacs	Previous cardiac operation (Norwood Stage I)
PREV_CARD_OP_CON_SUR_OTHER	STS Intermacs	Previous cardiac operation (congenital operation, other)
PREV_CARD_OP_CON_SUR_PA_BAND	STS Intermacs	Previous cardiac operation (PA Banding)
PREV_CARD_OP_CON_SUR_SEN_MSTRD	STS Intermacs	Previous cardiac operation (d- Transposition of the Great Vessels Repair – atrial switch (Senning/Mustard))
PREV_CARD_OP_CON_SUR_SEP_DEFCT	STS Intermacs	Previous cardiac operation (Complete AV Septal Defect Repair)
PREV_CARD_OP_CON_SUR_TOV_REP	STS Intermacs	Previous cardiac operation (TOV/DORV/RVOTO Repair)

© 2022 Cascino TM et al. *JAMA Network Open.*

PREV_CARD_OP_CON_SUR_TRUNCUS	STS Intermacs	Previous cardiac operation (Truncus Arteriosus Repair)
PREV_CARD_OP_CON_SUR_VSD_REP	STS Intermacs	Previous cardiac operation (VSD Repair)
PREV_CARDIAC_OPER_CON_CAR_SUR	STS Intermacs	Previous cardiac operation (Congenital card surgery)
PREV_CARDIAC_OPER_ANEURS_DOR	STS Intermacs	Previous cardiac operation (aneurysmectomy)
PREV_CARDIAC_OPER_AVR	STS Intermacs	Previous cardiac operation (aortic valve replacement/repair)
PREV_CARDIAC_OPER_CABG	STS Intermacs	Previous cardiac operation (coronary artery bypass grafting)
PREV_CARDIAC_OPER_MVR	STS Intermacs	Previous cardiac operation (mitral valve repair/replacement)
PREV_CARDIAC_OPER_TVR	STS Intermacs	Previous cardiac operation (Triscuspid replacement /repair)
PRIMARY_DGN	STS Intermacs	Primary diagnosis
PUL_DIA_PRES	STS Intermacs	Pulmonary artery diastolic pressure (mmHg)
PUL_SYS_PRES	STS Intermacs	Pulmonary artery systolic pressure (mmHg)
PUL_WEDGE_PRES	STS Intermacs	Pulmonary artery wedge pressure (mmHg)
PX_PROFILE	STS Intermacs	Patient profile at time of implant
RACE	STS Intermacs	Race
RVEF	STS Intermacs	Right ventricular ejection fraction
SGOT_AST	STS Intermacs	Aspartate Aminotransferase/AST (u/L)
SGPT_ALT		Alanine Aminotransferase/ALT (u/L)
SODIUM_MEQ_L	STS Intermacs	Sodium (mEq/L)
SYS_BP	STS Intermacs	Systolic blood pressure (mmHg)

© 2022 Cascino TM et al. *JAMA Network Open.*

WBC_X10_3_UL<br><br>WGT_KG	STS Intermacs<br><br>STS Intermacs	White blood cell count (x10/uL)<br><br>General hemodynamics weight (kg)
BSC	AHA	Bed size code
CITYRK_TOP25	AHA	City size rank (1-25, 26-50, 51-75, 76-100, greater than top 100)
COMMTY	AHA	Community Hospital flag
MAPP18	AHA	Critical Access Hospital
MAPP19	AHA	Rural Referral Center
MAPP3	AHA	Participating site recognized for one or more Accreditation Council for Graduate Medical Education accredited programs
MAPP5	AHA	Medical school affiliation reported to American Medical Association
SERV	AHA	Service code
CNTRL	AHA	Control code

© 2022 Cascino TM et al. *JAMA Network Open.*

eTable 2. Variables used for propensity matching

Variable Name	Original Location	Label
DEVICE_STRATEGY	STS Intermacs	Current device strategy
AGE	STS Intermacs	Age at implant
Gender	STS Intermacs	Male or female or unknown
BLOOD_TYPE	STS Intermacs	Blood type
RACE	STS Intermacs	Race
ETHNICITY	STS Intermacs	Ethnicity
BMI	STS Intermacs	Body mass index(kg/m2)
CREAT_MG_DL	STS Intermacs	Creatinine (mg/dL)
IMPL_YR	STS Intermacs	Implant year
HOSP_CTRL_UNOHOSP_INDX _UNOS_REGIONS_REGION	STS Intermacs	UNOS region of center
device_type	STS Intermacs	Device type (axial, hybrid magnetically levitated or fully magnetically levitated)
BMI	STS Intermacs	Body mass index(kg/m2)
PRIMARY_DGN	STS Intermacs	Primary diagnosis
Payor	STS Intermacs	Health insurance
CC2_PULMONARY_HYPERTENSION	STS Intermacs	Concern or contradiction that is a limitation for transplant listing (pulmonary hypertension)
CC2_ADVANCED_AGE_M	STS Intermacs	Concern or contradiction that is a limitation for transplant listing (advanced age)
CC2_LARGE_BMI	STS Intermacs	Concern or contradiction that is a limitation for transplant listing (large BMI)
CC2_OTHER_CO_MORBIDITY	STS Intermacs	Concern or contradiction that is a limitation for transplant listing (other comorbidity)
CC2_SEVERE_DIABETES	STS Intermacs	Concern or contradiction that is a limitation for transplant listing (severe diabetes)

© 2022 Cascino TM et al. *JAMA Network Open.*

CC2_Tobacco	STS Intermacs	Concern or contradiction that is a limitation for transplant listing (current smoking or history of smoking)
CC2_OtherDrugUse	STS Intermacs	Concern or contradiction that is a limitation for transplant listing (history of drug use or history of alcohol use or narcotic dependence)
CC2_psychosocial	STS Intermacs	Concern or contradiction that is a limitation for transplant listing (severe depression or other major psychiatric diagnosis or limited social support or limited cognitive understanding or repeated noncompliance)
CC2_PX_DOES_NOT_WANT_TX	STS Intermacs	Concern or contradiction that is a limitation for transplant listing (patient doesn't want a transplant)
CC2_fraility	STS Intermacs	Concern or contradiction that is a limitation for transplant listing (fraity or malnutrition/cachexia/musculoskeletal limitation)
CC2_malignancy_history		Concern or contradiction that is a limitation for transplant listing (history of lymphoma/leukemia or history of solid organ cancer)

© 2022 Cascino TM et al. *JAMA Network Open.*

eTable 3. Center characteristics of LVAD recipients

	Overall	LVAD-only	LVAD/Transplant	P-Value
**UNOS region (%)**				<0.001
Region 1	871 (3.9)	106 (3.4)	765 (4.0)	
Region 2	2523 (11.4)	532 (16.9)	1991 (10.4)	
Region 3	2559 (11.5)	365 (11.6)	2194 (11.5)	
Region 4	2213 (10.0)	69 (2.2)	2144 (11.2)	
Region 5	2188 (9.8)	393 (12.5)	1795 (9.4)	
Region 6	781 (3.5)	187 (5.9)	594 (3.1)	
Region 7	2344 (10.5)	61 (1.9)	2283 (12.0)	
Region 8	1446 (6.5)	174 (5.5)	1272 (6.7)	
Region 9	1647 (7.4)	481 (15.2)	1166 (6.1)	
Region 10	2442 (11.0)	282 (8.9)	2160 (11.3)	
Region 11	3207 (14.4)	506 (16.0)	2701 (14.2)	
**Number of beds (%)**				<0.001
50-99	88 (0.4)	88 (2.8)	0 (0.0)	
100-199	74 (0.3)	50 (1.6)	24 (0.1)	
200-299	750 (3.4)	169 (5.4)	581 (3.0)	
300-399	1209 (5.4)	466 (14.8)	743 (3.9)	
400-499	2894 (13.0)	196 (6.2)	2698 (14.2)	
500 or more	17206 (77.4)	2187 (69.3)	15019 (78.8)	
**Top 100 largest cities (%)**	15758 (70.9)	1479 (46.9)	14279 (74.9)	<0.001
**US City rank size (%)**				<0.001
1-25	8285 (37.3)	673 (21.3)	7612 (39.9)	
26-50	3680 (16.6)	332 (10.5)	3348 (17.6)	
51-75	2432 (10.9)	460 (14.6)	1972 (10.3)	
76-100	1361 (6.1)	14 (0.4)	1347 (7.1)	
Greater than top 100	6463 (29.1)	1677 (53.1)	4786 (25.1)	
**No ACGME accredited programs (%)**	1072 (4.8)	640 (20.3)	432 (2.3)	<0.001
**No medical school (%)**	693 (3.1)	546 (17.3)	147 (0.8)	<0.001
**Hospital authority (%)**				<0.001
Government, Nonfederal - State	2533 (11.4)	104 (3.3)	2429 (12.7)	
Government, Nonfederal - Hospital district	1003 (4.5)	51 (1.6)	952 (5.0)	
Not-for-profit Church	1762 (7.9)	473 (15.0)	1289 (6.8)	
Other not-for-profit	16355 (73.6)	2426 (76.9)	13929 (73.1)	
For-profit partnership	200 (0.9)	55 (1.7)	145 (0.8)	
For profit corporation	368 (1.7)	47 (1.5)	321 (1.7)	

Continuous variables are reported as medians (IQRs) and categorical variables are reported as and counts (%). UNOS, United Network for Organ Sharing; US, United States; ACGME, Accreditation Council for Graduate Medical Education.

© 2022 Cascino TM et al. *JAMA Network Open.*

eTable 4. Results of multivariable adjusted models for the interaction between transplant center status and BTT listing strategy

	Adjusted odds ratio*	95% confidence interval	P-Value
**Transplant volume (vs. none)**			
**<10**	1.48	1.05 – 2.08	0.024
**10-19**	1.78	1.32 – 2.39	<0.001
**20-29**	1.92	1.42 – 2.60	<0.001
**30-39**	1.63	1.18 – 2.26	0.003
**40-49**	1.64	1.16 – 2.30	0.005
**50-59**	1.80	1.24 – 2.63	0.002
**>60**	2.77	1.87 – 4.09	<0.001

*Adjusted for device type, age, body mass index, albumin, total bilirubin, right atrial pressure, race, implantable cardioverter defibrillator, New York Heart Association functional class, education level, marital status, payor, primary diagnosis, implant year, United Network for Organ Sharing region, hospital bed numbers, Accreditation Council for Graduate Medical Education program status, hospital authority, transplant limiting comorbidity status, prior coronary artery bypass graft surgery

© 2022 Cascino TM et al. *JAMA Network Open.*

eTable 5. Results of multivariable adjusted survival models for mortality with transplant as a competing risk

	Adjusted cause-specific hazard ratio*	95% confidence interval	P-Value
**LVAD/transplant center (vs. LVAD-only)**	0.99	0.90 – 1.08	0.79
*Adjusted for device strategy, device type, age, blood type, body mass index, right atrial pressure, race, implantable cardioverter defibrillator, ventilator during hospitalization, marital status, payor, primary diagnosis, implant year, United Network for Organ Sharing region, hospital bed numbers, US city rank size, hospital authority, transplant limiting comorbidity status, prior coronary artery bypass graft surgery Interaction.			

© 2022 Cascino TM et al. *JAMA Network Open.*

eTable 6. Results of multivariable adjusted survival models for the interaction between transplant center status and listing strategy and transplant

	Adjusted cause-specific hazard ratio*	95% confidence interval	P-Value
**LVAD/transplant center BTT (vs. LVAD-only)**	1.25	1.05 – 1.47	0.011
**LVAD/transplant center BTD (vs. LVAD-only)**	1.05	0.79 – 1.40	0.73
**LVAD/transplant center DT (vs. LVAD-only)**	1.63	1.31 – 2.04	<0.001

*Adjusted for device type, age, blood type, body mass index, right atrial pressure, race, implantable cardioverter defibrillator, ventilator during hospitalization, marital status, payor, primary diagnosis, implant year, United Network for Organ Sharing region, hospital bed numbers, US city rank size, hospital authority, transplant limiting comorbidity status, prior coronary artery bypass graft surgery Interaction. The P-Value for the interaction between transplant center status and BTT listing strategy and transplant was 0.036.

© 2022 Cascino TM et al. *JAMA Network Open.*

eTable 7. Results of multivariable adjusted survival models with inclusion of device strategy

	Adjusted cause-specific hazard ratio*	95% confidence interval	P-Value
**LVAD/transplant center (vs. LVAD-only)**	1.32	1.16 – 1.50	<0.001
*Adjusted for device strategy, device type, age, blood type, body mass index, right atrial pressure, race, implantable cardioverter defibrillator, ventilator during hospitalization, marital status, payor, primary diagnosis, implant year, United Network for Organ Sharing region, hospital bed numbers, US city rank size, hospital authority, transplant limiting comorbidity status, prior coronary artery bypass graft surgery Interaction.			

© 2022 Cascino TM et al. *JAMA Network Open.*

eTable 8. Results of multivariable adjusted survival models limited to patients under the age of 70 years

	Adjusted cause-specific hazard ratio*	95% confidence interval	P-Value
**LVAD/transplant center (vs. LVAD-only)**	1.29	1.11 – 1.46	<0.001
*Adjusted for device strategy, device type, age, blood type, body mass index, right atrial pressure, race, implantable cardioverter defibrillator, ventilator during hospitalization, marital status, payor, primary diagnosis, implant year, United Network for Organ Sharing region, hospital bed numbers, US city rank size, hospital authority, transplant limiting comorbidity status, prior coronary artery bypass graft surgery.			

© 2022 Cascino TM et al. *JAMA Network Open.*

eTable 9. Results of multivariable adjusted survival models not including right atrial pressure in the multivariable model

	Adjusted cause-specific hazard ratio*	95% confidence interval	P-Value
**LVAD/transplant center (vs. LVAD-only)**	1.32	1.6 – 1.50	<0.001
*Adjusted for device strategy, device type, age, blood type, body mass index, race, implantable cardioverter defibrillator, ventilator during hospitalization, marital status, payor, primary diagnosis, implant year, United Network for Organ Sharing region, hospital bed numbers, US city rank size, hospital authority, transplant limiting comorbidity status, prior coronary artery bypass graft surgery.			

© 2022 Cascino TM et al. *JAMA Network Open.*

eTable 10. Association of transplant center status with BTT-listing strategy using a P value cutoff of <.10 to select variables in the final multivariable model

	Adjusted odds ratio*	95% confidence interval	P-Value
**LVAD/transplant center (vs. LVAD-only)**	1.62	1.22 – 2.15	<0.001

*Adjusted for device type, age, blood type, gender, Intermacs® Patient Profile, race, body mass index, admitting diagnosis or planned admission, implantable cardioverter defibrillator, right ventricular ejection fraction, New York Heart Association functional class, albumin, total bilirubin, blood urea nitrogen, Aspartate Aminotransferase/AST, right atrial pressure, mitral valve replacement/repair within 48 hours, right ventricular assist device within 48 hours, ventilator within 48 hours, other cardiac surgery during the hospitalization, feeding tube during the hospitalization, intra-aortic balloon pump during the hospitalization, major myocardial infarction during the hospitalization, receipt of dopamine, receipt of norepinephrine, receipt of milrinone, education level, marital status, payor, primary diagnosis, implant year, United Network for Organ Sharing region, hospital bed numbers, Accreditation Council for Graduate Medical Education program status, hospital authority, transplant limiting comorbidity status, prior congenitally corrected transposition repair, prior aortic valve replacement/repair, prior coronary artery bypass graft surgery

© 2022 Cascino TM et al. *JAMA Network Open.*

eTable 11. Results of multivariable cause-specific Cox model for transplant in the 2 years following LVAD using a *P* value cutoff of <.10 to select variables in the final multivariable model

	Adjusted cause-specific hazard ratio*	95% confidence interval	P-Value
**LVAD/transplant center (vs. LVAD-only)**	1.31	1.16 – 1.49	<0.001

*Adjusted for device type, age, blood type, gender, Intermacs® Patient Profile, race, body mass index, implantable cardioverter defibrillator, heart rate, right ventricular ejection fraction, New York Heart Association functional class, systolic blood pressure, diastolic blood pressure, aortic regurgitation, albumin, pulmonary artery systolic pressure, pulmonary artery diastolic pressure, right atrial pressure, intra-aortic balloon pump within 48 hours, ventilator during the hospitalization, receipt of norepinephrine, receipt of milrinone, marital status, payor, primary diagnosis, implant year, United Network for Organ Sharing region, hospital bed numbers, US City rank size, Accreditation Council for Graduate Medical Education program status, hospital authority, transplant limiting comorbidity status, prior bi-directional Glenn, prior other congenital surgery, prior aortic valve replacement/repair, prior coronary artery bypass graft surgery

© 2022 Cascino TM et al. *JAMA Network Open.*

eTable 12. Results of multivariable cause-specific Cox model for transplant in the 2 years following LVAD among 6266 propensity matched recipients

	HR	95% CI		P-Value
**LVAD/transplant center (vs. LVAD-only)**	1.35	1.16	1.59	<0.001
**Device Type (vs axial)**				
Hybrid magnetically levitated	1.83	1.52	2.21	<0.001
Fully magnetically levitated	0.92	0.74	1.15	0.46
**Age (per year)**	0.97	0.97	0.98	<0.001
**Blood type (vs O)**				
A	1.71	1.46	2.00	<0.001
B	1.64	1.34	2.02	<0.001
AB	3.05	2.24	4.16	<0.001
**BMI (per kg/m^2)**	0.97	0.96	0.99	<0.001
**NYHA I-III (vs class IV)**	1.13	0.94	1.35	0.20
**Marital status (vs single)**				
Married	1.80	1.46	2.22	<0.001
Divorced/separated or other	1.64	1.29	2.08	<0.001
**Payor (vs. Medicare)**				
Medicaid	0.73	0.51	1.05	0.089
Commercial and HMO	1.60	1.26	2.03	<0.001
Other	1.42	1.14	1.77	0.002
**UNOS region (vs. region 1)**				
Region 2	0.68	0.47	0.98	0.038
Region 3	0.79	0.54	1.16	0.23
Region 4	0.46	0.24	0.88	0.018
Region 5	0.85	0.59	1.21	0.36
Region 6	1.45	0.95	2.22	0.089
Region 7	0.98	0.54	1.76	0.94
Region 8	1.15	0.71	1.88	0.57
Region 9	0.90	0.64	1.28	0.57
Region 10	0.67	0.45	0.99	0.047
Region 11	0.63	0.43	0.91	0.015
**Hospital bed numbers (vs. ≥500)**				
50-99	2.00	0.85	4.69	0.11
100-199	3.66	1.04	12.87	0.043
200-299	0.73	0.48	1.12	0.15
300-399	1.60	1.22	2.10	<0.001
400-499	1.41	1.13	1.75	0.002
**US City rank size (vs. 1-25)**				
26-50	0.60	0.47	0.78	<0.001
51-75	0.94	0.72	1.24	0.68
76-100	0.93	0.63	1.38	0.72
Greater than top 100	0.73	0.61	0.87	<0.001
**No medical school affiliation (vs. affiliation)**	0.39	0.26	0.58	<0.001
**Any transplant limiting comorbidity (vs. none)**	0.31	0.27	0.36	<0.001

© 2022 Cascino TM et al. *JAMA Network Open.*

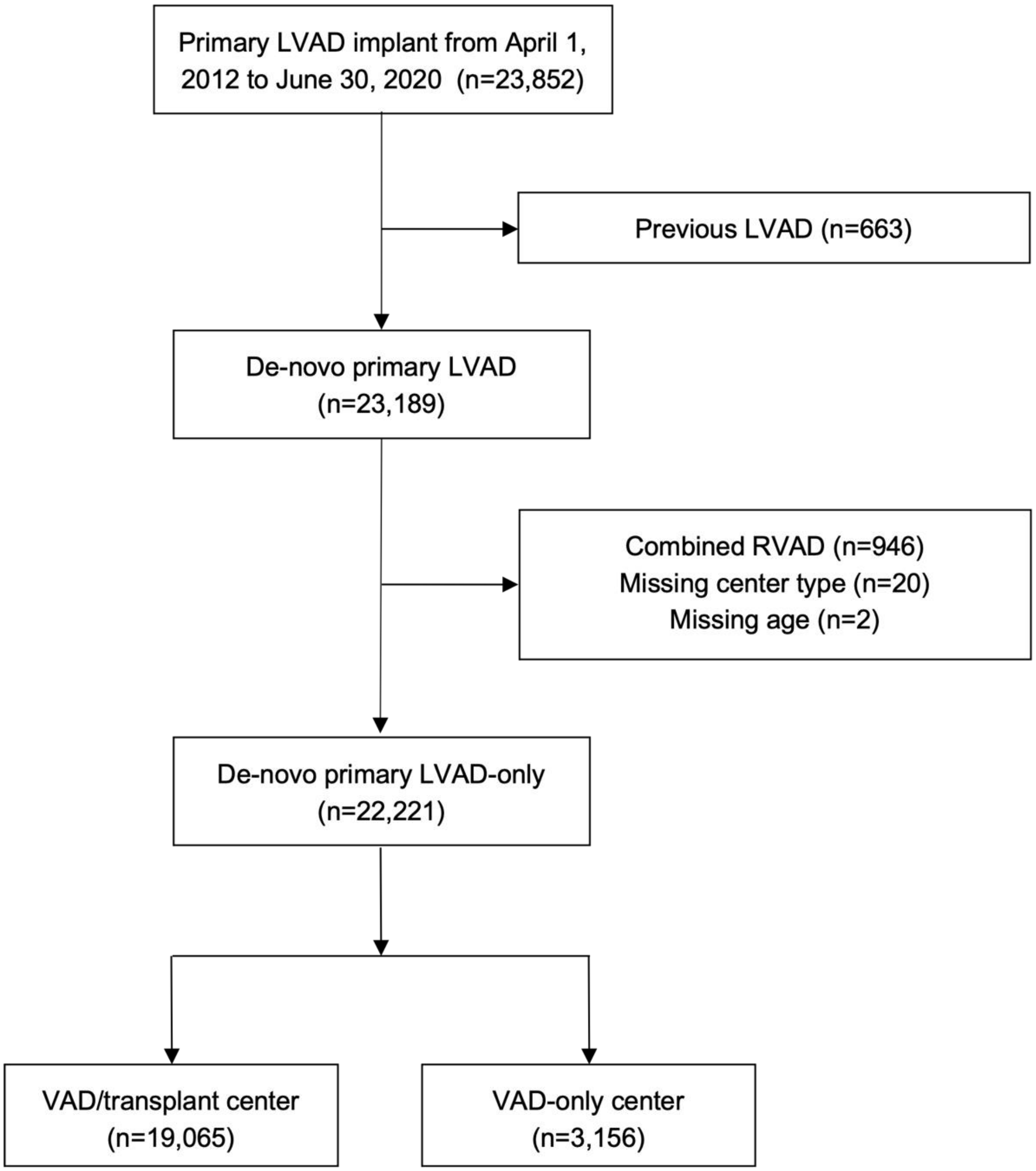

eFigure . Construction of cohort

© 2022 Cascino TM et al. *JAMA Network Open.*

eMethods 1. Additional information on The Society of Thoracic Surgeons (STS)-Intermacs database

The Society of Thoracic Surgeons (STS)-Intermacs registry was established in 2005 at the University of Alabama at Birmingham and was initially a joint effort between the National Heart, Lung, and Blood Institute, the Food and Drug Administration, and the Centers for Medicare and Medicaid Services to prospectively monitor outcomes for advanced heart failure patients who receive receiving a Food and Drug Administration-approved durable mechanical circulatory support device, including LVADs. Administration and oversight of the Intermacs registry was changed to The Society of Thoracic Surgeons in 2018. STS-Intermacs collects comprehensive demographic and clinical information for patients undergoing LVAD implantation. Patient consent to submit data to STS-Intermacs is determined by local hospital policy.

© 2022 Cascino TM et al. *JAMA Network Open.*

eMethods 2. Determination of therapeutic intent categorization

The groupings for BTT, BTC, and DT were created as follows from the INTERMACS assigned strategies: 1) BTT - bridge to transplant (listed) + bridge to transplant (likely) + bridge to recovery; 2) BTC - bridge to transplant (moderately likely) + bridge to transplant (unlikely) + rescue therapy + other; and 3) DT - destination therapy.13, 14 This categorization was chosen because of prior work documenting similar average transplant rates across patients within each of these groups (e.g., transplant rates for BTT (listed) + BTT (likely) similar).[1, 2]

eReferences

1. Teuteberg JJ, Stewart GC, Jessup M, et al. Implant strategies change over time and impact outcomes: insights from the INTERMACS (Interagency Registry for Mechanically Assisted Circulatory Support). *JACC Heart Fail*. Oct 2013;1(5):369-78. doi:10.1016/j.jchf.2013.05.006
2. Likosky DS, Yang G, Zhang M, et al. Interhospital variability in health care-associated infections and payments after durable ventricular assist device implant among Medicare beneficiaries. *J Thorac Cardiovasc Surg*. May 4 2021;doi:10.1016/j.jtcvs.2021.04.074

© 2022 Cascino TM et al. *JAMA Network Open.*
