# Supplementary material for: Comparison of Evaluations for Heart Transplant Before Durable Left Ventricular Assist Device and Subsequent Receipt of Transplant at Transplant vs Nontransplant Centers
Source: JAMA Netw Open. 2022 Nov 7;5(11):e2240646. doi: 10.1001/jamanetworkopen.2022.40646 (PMC9641540; doi:10.1001/jamanetworkopen.2022.40646)
Supplement: Supplement 2. — Nonauthor Collaborators. Michigan Congestive Heart Failure Investigators [file jamanetwopen-e2240646-s002.pdf]

Supplemental Online Content: Nonauthor Collaborators
*First name, last name, and suffix (if applicable) are required and will appear in PubMed.

***Group Name(s): The Michigan Congestive Heart Failure Investigators**

***First Name and Middle Initial(s)**	***Last Name**	***Suffix (eg, Jr, III)**	Academic Degrees	Institution	Location (city, state/province, country)	Role or Contribution, eg, chair, principal investigator	Group (if more than 1 Group listed in the byline) and/or Subgroup (eg, Steering Committee)
Khalil M	Nassar		BS	University of Michigan - Department of Cardiac Surgery	Ann Arbor, MI, USA	Collaborator, non-financial contributions, including interpreting the study findings and framing of the discussion	Non-author group collaborator (Michigan CHF Investigators Team)
Paul	Tang		MD, PhD	University of Michigan - Department of Cardiac Surgery	Ann Arbor, MI, USA	Collaborator, non-financial contributions, including interpreting the study findings and framing of the discussion	Non-author group collaborator (Michigan CHF Investigators Team)
Grace	Chung		PhD	University of Michigan	Ann Arbor, MI, USA	Collaborator, non-financial contributions, including interpreting the study findings and framing of the discussion	Non-author group collaborator (Michigan CHF Investigators Team)

Supplemental Online Content: Nonauthor Collaborators
*First name, last name, and suffix (if applicable) are required and will appear in PubMed.

***First Name and Middle Initial(s)**	***Last Name**	***Suffix (eg, Jr, III)**	Academic Degrees	Institution	Location (city, state/province, country)	Role or Contribution, eg, chair, principal investigator	Group (if more than 1 Group listed in the byline) and/or Subgroup (eg, Steering Committee)
Tessa	Watt		MD, MSc	University of Michigan	Ann Arbor, MI, USA	Collaborator, non-financial contributions, including interpreting the study findings and framing of the discussion	Non-author group collaborator (Michigan CHF Investigators Team)
Michael P	Thompson		PhD	University of Michigan - Department of Cardiac Surgery	Ann Arbor, MI, USA	Collaborator, non-financial contributions, including interpreting the study findings and framing of the discussion	Non-author group collaborator (Michigan CHF Investigators Team)
Allison M	Janda		MD	University of Michigan - Department of Anesthesiology	Ann Arbor, MI, USA	Collaborator, non-financial contributions, including interpreting the study findings and framing of the discussion	Non-author group collaborator (Michigan CHF Investigators Team)
